# Preoperative weight loss program involving a 20‐day very low‐calorie diet for obesity before laparoscopic gastrectomy for gastric cancer

**DOI:** 10.1111/ases.12479

**Published:** 2018-03-25

**Authors:** Kentaro Inoue, Sawako Yoshiuchi, Mika Yoshida, Natsuko Nakamura, Sachiko Nakajima, Akiko Kitamura, Keiko Mouri, Taku Michiura, Hiromi Mukaide, Takashi Ozaki, Hirokazu Miki, Hiroaki Yanagimoto, Sohei Satoi, Masaki Kaibori, Madoka Hamada, Yutaka Kimura, Masanori Kon

**Affiliations:** ^1^ Department of Surgery Kansai Medical University Hirakata Japan; ^2^ Health Science Center Kansai Medical University Hospital Hirakata Japan; ^3^ Department of Gastrointestinal Surgery Kansai Medical University Hospital Hirakata Japan

**Keywords:** Gastrectomy, obesity, weight loss programs

## Abstract

**Introduction:**

The increased visceral fat in patients with obesity can increase the technical difficulty of surgery. This study was performed to evaluate a preoperative 20‐day very low‐calorie diet for obesity before laparoscopic gastrectomy for gastric cancer.

**Methods:**

This prospective single‐center study involved patients with obesity who were planning to undergo laparoscopic gastrectomy for gastric cancer. Obesity was defined according to the Japanese criteria: BMI ≥25 kg/m^2^ or waist circumference ≥85 cm in men and ≥90 cm in women. The patients underwent a preoperative 20‐day very low‐calorie diet and received nutritional counseling. Weight loss, body composition, visceral fat mass, and operative outcomes were evaluated.

**Results:**

Thirty‐three patients were enrolled from September 2013 to August 2015. Their median age was 71 years, and 78.8% were men. Their median bodyweight and BMI were 72.3 kg (range, 53.8–82.5 kg) and 26.0 kg/m^2^ (range, 23.5–31.0 kg/m^2^), respectively. The patients achieved a mean weight loss of 4.5% (95% confidence interval [CI]: 3.8–5.1), corresponding to 3.2 kg (95%CI: 2.7–3.7 kg). Body fat mass was significantly decreased by a mean of 2.5 kg (95%CI: 1.9–3.1), whereas skeletal muscle mass was unaffected (mean: −0.20 kg [95%CI: −0.55–0.15]). The visceral fat mass reduction rate was high as 16.8% (range, 11.6%–22.0%). All patients underwent laparoscopic gastrectomy as planned. Severe postoperative morbidity (Clavien–Dindo grade ≥III) was seen in only one patient (3.0%).

**Conclusion:**

The preoperative 20‐day very low‐calorie diet weight loss program is promising for the treatment of obesity before laparoscopic gastrectomy for gastric cancer.

## Introduction

Much of the research on surgical treatment of gastric cancer has focused on obesity in recent years 1, 2, 3, 4, 5, 6, 7, 8, 9, 10, 11, 12, 13. Obesity is associated with increased blood loss, a prolonged operative time, a high rate of postoperative complications, and inadequate lymph node staging. The characteristically increased visceral fat in patients with obesity can make surgical procedures more difficult, especially complex oncological procedures such as laparoscopic gastrectomy for gastric cancer.

The World Health Organization defined obesity as a BMI ≥30 kg/m^2^ 14. Based on the available data in Asia, the World Health Organization Expert Consultation concluded that Asians generally have a higher percentage of body fat than white people with the same BMI 15. Therefore, in Japan, obesity is defined as a BMI ≥25 kg/m^2^ 16. Furthermore, the 2014 Japanese National Health and Nutrition Survey reported a considerably high percentage of obesity (BMI ≥25 kg/m^2^) among Japanese men and women aged ≥20 years (28.7% and 21.3%, respectively) 17.

A preoperative weight loss program is recommended by most bariatric surgical centers 18, 19. Weight loss interventions reportedly decrease visceral fat and liver volumes 20, 21. Excess visceral fat and a high liver volume are known to complicate the technical aspects of bariatric surgery because they can increase the blood loss volume, operating time, and risk of complications. Preoperative weight loss programs usually involve a very low‐calorie diet (VLCD) with a total caloric intake <1100 kcal/day for about 4 weeks before surgery 22. Some VLCD are specifically formulated with a lower carbohydrate and fat content to encourage fat loss through ketosis and a high protein content to preserve lean body mass. Commercially available VLCD are associated with minimal adverse effects and dropout rates. Most VLCD studies have reported a 5% to 10% total weight loss and 10% liver volume reduction 22.

We hypothesized that VLCD for patients with obesity before laparoscopic gastrectomy for gastric cancer could help these patients to achieve sufficient weight loss that would translate into a shorter operative time, less blood loss, fewer complications, and more accurate lymph node staging. However, the potentially negative effects of such programs include a longer preoperative period, patient discomfort, and increased morbidity associated with the performance of a surgical procedure after loss of lean body mass (especially skeletal muscle mass). Therefore, we evaluated the efficacy and safety of a preoperative weight loss program involving a 20‐day VLCD for patients with obesity undergoing laparoscopic gastrectomy for gastric cancer.

## Materials and Methods

The study was approved by the institutional review board of Kansai Medical University Hospital. The study was registered in the UMIN Clinical Trials Registry under the identifier UMIN000009691. Based on the ethical principles outlined in the Declaration of Helsinki, all patients provided written informed consent before enrollment 23.

### Patients

This prospective single‐center study involved patients with obesity who were planning to undergo laparoscopic gastrectomy for gastric cancer (cT1N0‐1M0 or cT2N0M0). Obesity was defined according to the Japanese criteria: BMI ≥25 kg/m^2^ or waist circumference ≥85 cm in men and ≥90 cm in women 16. All patients were aged ≥20 years, had an ASA physical status of I or II, and had adequate organ function. Patients with a history of upper abdominal open surgery, uncontrolled diabetes, active infectious disease, and treatment with steroids were excluded. Patients with a history of allergic reactions to soybeans, milk, eggs, peanuts, or gelatin were excluded because these foods were present in the VLCD.

### Weight loss program and VLCD preparation

The preoperative weight loss program was performed in an outpatient setting. At our hospital, the waiting period for laparoscopic gastrectomy of gastric cancer was usually about 1 month. During the preoperative waiting period, eligible patients received nutritional counseling from a dietitian. Patients received information on a nutritionally balanced diet, low‐calorie foods, and appropriate mealtimes. The patients started the VLCD (ObeCure®; USCure, Tokyo, Japan) 20 days before surgery, and one follow‐up visit with the dietitian was planned during the weight loss program. The VLCD contained a total of 178 kcal, 15‐g carbohydrate, 22‐g protein, 2‐g fat, and the recommended daily intake levels of vitamins and minerals. The VLCD was provided in powder form to be mixed with water. Each day, one VLCD sachet was used to replace one of three meals (breakfast, lunch, or dinner) during the 20‐day program. In addition to the VLCD, patients were allowed to eat low‐calorie vegetables at the replaced mealtime. We recommended that the patients replace dinner with the VLCD sachet, and most did so; however, some patients replaced lunch with the VLCD sachet because lunch was their main meal of the day.

Because the optimal duration of a preoperative VLCD has yet to be defined, we selected a 20‐day program to achieve the largest possible effect with consideration of the waiting period for surgery at our hospital. Patients who discontinued the VLCD for any reason were managed with nutritional counseling. The VLCD preparation was free of charge for the study patients.

### Surgical procedure

Laparoscopic gastrectomy was performed according to the guideline of the Japanese Gastric Cancer Association 24. The standard procedures were Roux‐en‐Y reconstruction after distal gastrectomy and total gastrectomy as well as esophagogastrostomy after proximal gastrectomy. All laparoscopic procedures were performed either by a surgeon qualified by the Endoscopic Surgical Skill Qualification System of the Japan Society for Endoscopic Surgery or with the certified surgeon acting as the lead assistant. Intravenous antibiotic prophylaxis was given to all patients preoperatively and intraoperatively, and low‐molecular‐weight heparin was used as postoperative antithrombotic prophylaxis for 7 days.

### Monitoring

The primary study end‐point was the percentage of weight loss. The weight change was calculated as the difference between the weight at the time of the first counseling session with the dietician and the preoperative weight after conclusion of the weight loss program (1–3 days before surgery). Weight reduction is described as the relative difference in the percentage of total body weight.

Secondary end‐points included evaluation of changes in body composition using a body composition analyzer (InBody 770®; InBody, Seoul, Korea) and changes in abdominal subcutaneous and visceral fat mass by one cross‐sectional CT scan of the umbilical area. Fat mass areas were identified by a surgeon blinded to the study data, and the areas were calculated by image analysis software (Data Picker ver.1.2; Blue Moon Factory, Tokyo, Japan). The maximum areas of the lateral segment of the liver were measured by the same method. Other secondary end‐points included evaluation of tolerability of the VLCD, changes in biochemical parameters, operative morbidity, operative time, and blood loss. Self‐evaluated side‐effects and compliance with the VLCD were recorded at each consultation with the dietician and surgeon. Blood samples were collected after an overnight fast and analyzed for glycated hemoglobin (HbA1c), glucose, insulin, albumin, aspartate aminotransferase, gamma‐glutamyl transpeptidase, triglycerides, low‐density lipoprotein, and homeostasis model assessment–insulin resistance. Homeostasis model assessment–insulin resistance was calculated according to methods used Matthews *et al*. as follows 25: fasting insulin (U/L) × fasting glucose (mg/dL) / 405. Postoperative morbidities were graded using the Clavien–Dindo classification 26. Gastric cancer staging was performed according to the third English edition of the Japanese Classification of Gastric Carcinoma 27.

### Comparative analysis with historical controls

To evaluate the efficacy of preoperative weight loss for laparoscopic gastrectomy, the surgical outcomes were compared with those of patients with obesity (BMI ≥25 kg/m^2^) who underwent laparoscopic surgery without a weight loss program from June 2009 to October 2011 at our institute. Because laparoscopic proximal gastrectomy, total gastrectomy, and pylorus‐preserving gastrectomy were not performed from June 2009 to October 2011, we analyzed the outcomes of patients who underwent laparoscopic distal gastrectomy with Roux‐en‐Y reconstruction. The lymph node dissection and reconstruction techniques were the same in both study periods (June 2009–August 2015).

### Sample size and statistical analysis

The percentage of weight loss that is safe and effective for the surgical treatment of gastric cancer remains unclear. We expected weight loss at the time of surgery to be >3% because a modest weight loss of 3% is known to improve obesity‐related risk factors or conditions in Japanese patients with obesity 28. We planned to enroll 33 patients with an alpha level of 0.05, power of 75%, standard deviation of 6%, and missing data rate of 10%. All analyses were intention‐to‐treat analyses. Some data were not examined at one of the evaluation time points; these data were excluded from the analysis (CT scan in two cases, body composition analysis in two cases, HbA1c in one case, insulin in two cases, and triglycerides in three cases).

Data are presented as median and range or mean with 95% confidence interval as dispersion descriptive measures. Continuous variables were analyzed using a paired *t*‐test to compare variables from before and after the weight loss program. In the comparative analysis with the historical controls, continuous variables were analyzed using the Wilcoxon test, and qualitative variables were analyzed using the χ^2^ test. Data were considered statistically significant when *P* <0.05 for a two‐tailed distribution. All statistical analyses were performed using statistical discovery software (JMP version 11.2 for Windows; SAS Institute Inc., Cary, USA).

## Results

From September 2013 to August 2015, 33 patients were enrolled in this study. The patients’ baseline characteristics are shown in Table 1. The patients had a median age of 71 years (range, 41–81 years), and 78.8% were men. All patients were Asian. The median body weight and BMI were 72.3 kg (range, 53.8–82.5 kg) and 26.0 kg/m^2^ (range, 23.5–31.0 kg/m^2^), respectively. Of the 33 patients, 24 were taking one or more medications for hypertension, hyperlipidemia, diabetes, or thrombosis. One patient stopped the VLCD after 10 days because the taste was not tolerable. The compliance rate was 96.9% for all patients receiving the VLCD. At the study end, no adverse events associated with the VLCD were reported.

**Table 1:** Baseline demographic and clinical characteristics of patients (*n* = 33)

Characteristics	
Age (years), median (range)	71 (41–81)
Sex, *n* (%)	
Female	7 (21.2)
Male	26 (78.8)
Race	
Asian	33 (100.0)
Bodyweight (kg), median (range)	72.3 (53.8–82.5)
BMI (kg/m^2^), median (range)	26.0 (23.5–31.0)
ASA‐PS, *n* (%)	
Class 1	9 (27.3)
Class 2	23 (69.7)
Medications, *n* (%)	
None	9 (27.3)
Hypertension	24 (72.7)
Hyperlipidemia	6 (18.2)
Diabetes	5 (15.2)
Thrombosis	4 (12.1)

ASA‐PS, American Society of Anesthesiologists physical status.

### Changes in body weight, BMI, body composition, and abdominal fat

**Figure 1:**
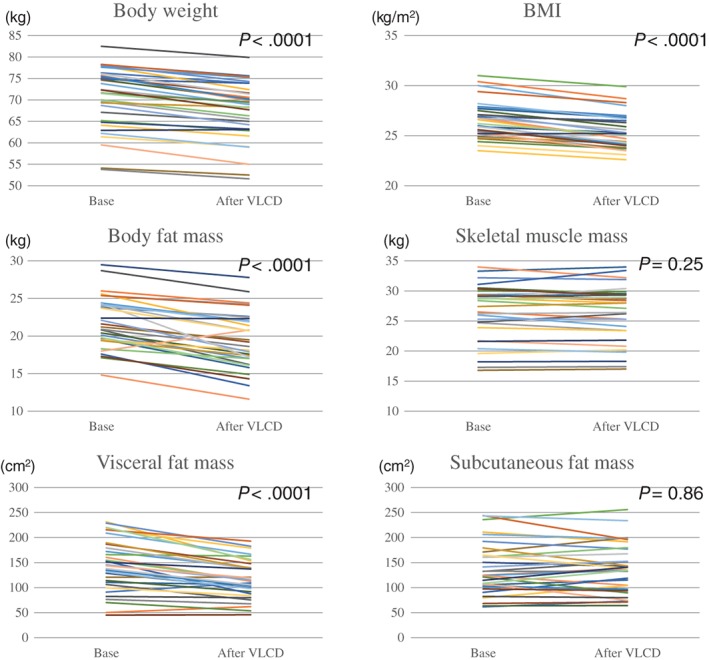
Changes in body weight, BMI, body composition, and abdominal fat. VLCD, very low‐calorie diet.

Figure 1 shows the results obtained using the preoperative weight loss program. The primary end‐point was the percent of weight loss by the 20‐day VLCD program. The mean weight loss was 4.5% (95% confidence interval [CI]: 3.8–5.1), corresponding to a weight reduction of 3.2 kg (95%CI: 2.7–3.7) (*P* < 0.0001). As a result, weight reduction was observed in virtually all cases. The mean BMI decreased significantly from 26.5 to 25.3 kg/m^2^; the mean BMI loss was 1.2 kg/m^2^ (95%CI: 1.0–1.4). Changes in body composition were measured with a body composition analyzer and CT scan. A significant reduction in body fat mass was observed in the body composition analysis (*P* < 0.0001). The mean reduction in body fat mass was 2.5 kg (95%CI: 1.9–3.1). The mean change in skeletal muscle mass was only −0.20 kg (95%CI: −0.55–0.15). In the abdominal fat analysis by CT, visceral fat mass decreased significantly from 146.5 to 118.4 cm^2^ (*P* < 0.0001); the reduction rate was as high as 16.8% (range, 11.6%–22.0%). Because the subcutaneous fat mass was minimal (−1.4% [95%CI: −7.3–4.5]), weight loss was mainly attributed to the loss of visceral fat mass.

### Waist circumference, waist‐to‐hip ratio, biochemical parameters, and liver volume

Table 2 shows the results of the secondary end‐points during the preoperative weight loss program. Waist circumference and waist‐to‐hip ratio were significantly decreased (*P* < 0.0001). The baseline biochemical parameters were within normal limits. However, reasonably good changes were obtained in the HbA1c and triglyceride concentrations. Inconsistent with other reports of bariatric patients, a decrease in the volume of the lateral segment of the liver was not observed in this population.

**Table 2:** Changes in anthropometric and biochemical parameters after VLCD (*n* = 33)

Outcomes	Mean at baseline	Mean after VLCD	Mean change	95%CI	*P*‐value
Waist circumference (cm)	92.7	89.9	−2.74	−3.30–−2.19	<0.0001
Waist‐to‐hip ratio	0.97	0.96	−0.01	−0.017–−0.008	<0.0001
HbA1c (NGSP) (%)	6.01	5.88	−0.13	−0.24–−0.02	0.02
Glucose (mg/dL)	109.9	103.2	−6.67	−17.1–3.73	0.90
HOMA‐IR	3.54	3.13	−0.41	−1.79–0.97	0.73
ALB (g/dL)	4.34	4.42	+0.08	−0.03–0.18	0.16
AST (IU/L)	23.0	22.9	−0.09	−3.17–2.99	0.95
GGTP (IU/L)	36.0	31.5	−4.48	−15.1–6.13	0.40
TG (mg/dL)	145.5	109.0	−36.5	−67.7–−5.39	0.02
LDL (mg/dL)	117.8	118.9	+1.13	−11.9–14.1	0.96
Lateral segment of liver (cm^2^)	30.3	30.4	+0.05	−1.51–1.61	0.95

ALB, albumin; AST, aspartate aminotransferase; CI, confidence interval; GGTP, gamma‐glutamyl transpeptidase; HOMA‐IR, homeostasis model assessment‐insulin resistance; LDL, low density lipoprotein; NGSP, National Glycohemoglobin Standardized Program; TG, triglyceride; VLCD, very low‐calorie diet.

### Surgical outcomes

All patients underwent laparoscopic gastrectomy as planned. No conversions to an open procedure occurred. The surgical outcomes are summarized in Table 3. The median operative time was 345 min, and the median blood loss was 50 mL. Most tumors were classified as pStage I. Postoperative morbidity occurred in 10 patients (30.3%), and a severe complication (grade ≥III) was seen in only one patient (3.0%). This patient required radiological intervention for gastroparesis. The most common morbidity was a pancreatic fistula (4 patients, 12.1%). All patients recovered without surgical or radiological interventions.

**Table 3:** Surgical outcomes (*n* = 33)

Outcomes	
Procedures, *n* (%)	
Total gastrectomy with Roux‐en‐Y reconstruction	3 (9.1)
Distal gastrectomy with Roux‐en‐Y reconstruction	27 (81.8)
Pylorus preserving gastrectomy	2 (6.1)
Proximal gastrectomy with esophagogastrostomy	1 (3.0)
Operative time (min), median (range)	345 (196–567)
Blood loss (mL), median (range)	50 (1–582)
Blood transfusion, *n* (%)	0 (0.0)
R0 surgery, *n* (%)	33 (100.0)
Lymph node dissection, *n* (%)	
D1+	26 (78.8)
D2	7 (21.2)
Retrieved nodes (*n*), median (range)	27 (7–47)
pStage, *n* (%)	
I	27 (81.8)
II	3 (9.1)
III	3 (9.1)
IV	0 (0.0)
Hospital mortality, *n* (%)	0 (0.0)
Postoperative morbidity, *n* (%)	10 (30.3)
Anastomotic leak	1 (3.0)
Pancreatic fistula	4 (12.1)
Anastomotic hemorrhage	1 (3.0)
Gastroparesis	1 (3.0)
Remote infection	2 (6.1)
Hepatobiliary disorder	1 (3.0)
Clavien–Dindo grade I/II/IIIA/IIIB/IV/V (*n*)	3/6/1/0/0/0

### Comparative analysis with historical controls

The comparative analysis with the historical controls is summarized in Table 4. Patient characteristics were well balanced in terms of age, sex, BMI, and pStage between the groups. There were no significant differences in the number of retrieved lymph nodes or operative time; however, the weight loss program was associated with an increase of three lymph nodes and a reduction in the operative time of 19 min. The blood loss volume was significantly smaller with than without the weight loss program (49 vs 76 mL, *P* = 0.043). There were no differences in postoperative morbidity. Postoperative morbidities were few even without the preoperative weight loss program (2 cases of grade II pancreatic fistula, 1 case of grade I gastroparesis, 1 case of grade II cardiac disease, and 1 case of grade IIIb hiatal hernia).

**Table 4:** Comparative analysis with historical controls†

	Historical controls (*n* = 23)	Present study (*n* = 27)	*P*‐value
	Without weight loss program	With weight loss program	
Period	June 2009–October 2011	September 2013–August 2015	
Procedure (*n*)			
LDGRY	23	27	
Age (years), median (range)	72 (46–83)	71 (40–81)	0.23
Sex, *n* (%)			0.31
Male	21 (91.3)	22 (81.5)	
Female	2 (8.7)	5 (18.5)	
BMI (kg/m^2^), median (range)	26.1 (25.0–31.9)	26.6 (23.5–31.0)	0.96
pStage, *n* (%)			0.32
I	21 (91.3)	22 (81.5)	
II	2 (8.7)	2 (7.4)	
III	0	3 (11.1)	
IV	0	0	
Lymph node dissection, *n* (%)			0.50
D1+	15 (65.2)	20 (74.1)	
D2	8 (34.8)	7 (25.9)	
Retrieved nodes (*n*), median (range)	23 (8–48)	26 (7–47)	0.32
Operative time (min), median (range)	374 (258–482)	355 (196–567)	0.35
Blood loss (mL), median (range)	76 (34–914)	49 (1–282)	0.043
Postoperative morbidity, *n* (%)	5 (21.7)	7 (25.9)	0.68
Clavien–Dindo grade I/II/IIIA/IIIB/IV/V (*n*)	1/3/0/1/0/0	1/5/1/0/0/0	

† The historical controls are obese patients (BMI ≥25 kg/m^2^) who underwent laparoscopic distal gastrectomy with Roux‐en‐Y reconstruction (LDGRY) without weight loss program between June 2009 and October 2011 at our institute.

## Discussion

Our study has shown that it is possible to achieve a preoperative mean weight loss of 4.5% (3.2 kg) and a 16.8% decrease in visceral fat using a short‐term (20‐day) VLCD program for patients with obesity before laparoscopic gastrectomy for gastric cancer. Theoretically, an aggressive reduction in the preoperative dietary intake could be associated with loss of skeletal muscle mass. In the present study, however, skeletal muscle mass was unaffected by the 20‐day VLCD program. To the best of our knowledge, this is the first study to investigate the effectiveness of a preoperative VLCD in patients with obesity undergoing gastric cancer surgery.

Weight loss achieved through conventional dietary and lifestyle changes can be difficult for patients, especially within a limited preoperative time period. To date, one prospective study has investigated the effects of a preoperative intervention for patients with obesity (BMI ≥25 kg/m^2^) undergoing gastric cancer surgery. Cho *et al*. evaluated a 4‐week training program involving aerobic exercise (treadmill, bicycle ergometer, swimming, dancing, or jogging), resistance training, and stretching 29. Their program successfully achieved a 1.34‐kg weight loss and a 15.7% decrease in visceral fat. Although their program was promising, it may be difficult for some older patients to participate in such exercise training. It should be noted that the patients in the study by Cho *et al*. were 8 years younger than those in our study 29, and the aging population continues to grow in Japan.

The use of a preoperative weight loss program has been widely adopted by most bariatric surgical centers 18, 19. Beneficial effects of such programs on liver volume, visceral and subcutaneous fat mass, and comorbidities have been demonstrated 20, 21, 30. One possible explanation for the beneficial effects on intraoperative and postoperative outcomes might be the reduced technical complexity associated with the reduced visceral fat and liver volume. Similar effects were found in a prospective observational study and a randomized controlled trial involving a 2‐week VLCD before elective laparoscopic cholecystectomy for patients with obesity 31, 32. Liver size reduction was not observed in the present study. Reductions in liver size as measured by imaging techniques after weight loss have been reported 21. This disparity might be attributable to the different baseline BMI of patients undergoing surgical treatment of gastric cancer versus bariatric surgery.

Another possible explanation could be the improved insulin sensitivity induced by weight loss. This effect could improve glucose control postoperatively, which has been demonstrated to reduce the risk of infectious complications. The higher rate of complications associated with obesity in surgery for gastric cancer is mainly attributed to an increase in the rate of surgical‐site infections 1, 2, 4, 5, 10, 13. The significantly reduced HbA1c concentration in our study supports the concept that preoperative weight loss programs may reduce postoperative infectious complications. Additionally, patients with obesity usually have comorbidities. Of our 33 patients, 24 were taking one or more medications for hypertension, hyperlipidemia, diabetes, or thrombosis. A modest weight loss of 3% is known to improve obesity‐related risk factors or conditions in Japanese patients with obesity 28. Therefore, preoperative weight loss programs may lead to a decrease in cardiovascular, thromboembolic, and pulmonary complications both during and after surgery.

The comparative analysis with the historical controls showed no differences in postoperative morbidities. The rate of postoperative morbidities was low even without a preoperative weight loss program. Surgeons make a great effort to avoid postoperative morbidity for every patient, which leads to low morbidity in patients with obesity without preoperative weight loss. However, such surgical procedures are very stressful for surgeons. Therefore, further research will be necessary to identify any advantages of a preoperative weight loss program in patients with obesity undergoing surgery for gastric cancer.

This study had several limitations, including its small sample size, single‐center design, and absence of an appropriate control group. In the comparative analyses with the historical controls, slightly better outcomes were achieved in the number of retrieved lymph nodes, operative time, and blood loss in the present study. There was an increase of three retrieved lymph nodes (*P* = 0.32), an operative time reduction of 19 min (*P* = 0.35), and an operative blood loss reduction of 27 mL (*P* = 0.043) in association with the preoperative weight loss program. These improvements were very small and may not be clinically meaningful. However, some changes occurred in the study period that likely affected the outcomes. The most important point is that not all procedures were completed by a single surgeon. More laparoscopic procedures were performed by young surgeons in patients who underwent the VLCD program (data not recorded). This change during the study period likely affected the small improvement related to the VLCD program.

This trial was also limited by the fact that the results may not be directly applicable to patients who do not receive nutritional counseling and encouragement from an experienced health professional. Nutritional counseling is an important aspect of the treatment of obesity.

The long operative time and low number of retrieved lymph nodes were limitations of this study. In a study by Oki *et al*. 33, the mean operative time and number of retrieved lymph nodes during laparoscopic distal gastrectomy in Asian patients with a BMI ≥25 kg/m^2^ were 321.9 min and 30.8, respectively. In a study by Sugimoto *et al*., these figures were 212 min and 29 6. Our operative time was longer, and the number of retrieved lymph nodes was lower than in these previous reports. These results suggest that our surgical technique is not yet mature and that surgeons with greater skills may not need their patients to undergo a preoperative weight loss program.

In conclusion, virtually all of the patients with obesity who underwent the 20‐day VLCD preoperative weight loss program in the present study achieved considerable weight loss without losing skeletal muscle mass. The weight loss induced by the program was a result of a considerable reduction in visceral fat mass. This 20‐day VLCD preoperative weight loss program seems to be promising for patients with obesity who are undergoing laparoscopic gastrectomy for gastric cancer. Prospective randomized controlled trials are warranted to clarify the advantages of the 20‐day VLCD preoperative weight loss program.
